# A family carer decision support intervention for people with advanced dementia residing in a nursing home: a study protocol for an international advance care planning intervention (mySupport study)

**DOI:** 10.1186/s12877-022-03533-2

**Published:** 2022-10-26

**Authors:** Andrew J E Harding, Julie Doherty, Laura Bavelaar, Catherine Walshe, Nancy Preston, Sharon Kaasalainen, Tamara Sussman, Jenny T van der Steen, Nicola Cornally, Irene Hartigan, Martin Loucka, Karolina Vlckova, Paola Di Giulio, Silvia Gonella, Kevin Brazil, On behalf of the mySupport Study Group, Wilco P. Achterberg, Mandy Visser, Catherine Buckley, Serena Fitzgerald, Tony Foley, Siobhan Fox, Alan Connolly, Ronan O’Caoimh, Selena O’Connell, Catherine Sweeney, Suzanne Timmons, Christine Brown Wilson, Gillian Carter, Emily Cousins, Kay De Vries, Josie Dixon, Karen Harrison Dening, Catherine Henderson, Adrienne McCann

**Affiliations:** 1 Division of Health Research, Faculty of Health and Medicine, Lancaster University, Lancaster, UK; 2 School of Nursing and Midwifery, Queens University Belfast, Belfast, UK; 3 Leiden University Medical Centre, Leiden University, Leiden, The Netherlands; 4 School of Nursing, McMaster University, Hamilton, Canada; 5 School of Social Work, McGill University, Hamilton, Canada; 6 School of Nursing and Midwifery, University College Cork, Cork, Republic of Ireland; 7 Centre for Palliative Care, Prague, Czech Republic; 8 Department of Clinical and Biological Sciences, University of Torino, Turin, Italy; 9 School of Nursing and Midwifery, Queens University Belfast, Belfast, UK

**Keywords:** Advance care planning, Nursing homes, Training, Palliative care, Implementation, Dementia

## Abstract

**Background:**

Where it has been determined that a resident in a nursing home living with dementia loses decisional capacity, nursing home staff must deliver care that is in the person's best interests. Ideally, decisions should be made involving those close to the person, typically a family carer and health and social care providers. The aim of the Family Carer Decisional Support intervention is to inform family carers on end-of-life care options for a person living with advanced dementia and enable them to contribute to advance care planning. This implementation study proposes to; 1) adopt and apply the intervention internationally; and, 2) train nursing home staff to deliver the family carer decision support intervention.

**Methods:**

This study will employ a multiple case study design to allow an understanding of the implementation process and to identify the factors which determine how well the intervention will work as intended. We will enrol nursing homes from each country (Canada *n =* 2 Republic of Ireland = 2, three regions in the UK *n =* 2 each, The Netherlands *n =* 2, Italy *n =* 2 and the Czech Republic *n =* 2) to reflect the range of characteristics in each national and local context. The RE-AIM (reach, effectiveness, adoption, implementation, maintenance) framework will guide the evaluation of implementation of the training and information resources. Our mixed methods study design has three phases to (1) establish knowledge about the context of implementation, (2) participant baseline information and measures and (3) follow up evaluation.

**Discussion:**

The use of a multiple case study design will enable evaluation of the intervention in different national, regional, cultural, clinical, social and organisational contexts, and we anticipate collecting rich and in-depth data. While it is hoped that the intervention resources will impact on policy and practice in the nursing homes that are recruited to the study, the development of implementation guidelines will ensure impact on wider national policy and practice. It is our aim that the resources will be sustainable beyond the duration of the study and this will enable the resources to have a longstanding relevance for future advance care planning practice for staff, family carers and residents with advanced dementia.

## Background

The progression of dementia can vary markedly between individuals, but is usually described in stages (mild, moderate, severe), where health deterioration worsens over time. Depending on the cause of dementia the early stage may occur within the first two years following diagnosis, the middle stage between the second to fourth following diagnosis and the final stage (advanced) in the years thereafter. Life expectancy for this terminal condition ranges from 3 – 10 years [1, 2]. It is common for people living with dementia to be admitted into a nursing home when they are in the moderate/advanced stage of dementia [2].

Where it has been determined that a person living with dementia lacks decisional capacity the nursing home staff must deliver care that is in the person's best interests. This is known as 'best interest decision-making '. Ideally, decisions and advance care plans should be made involving those close to the person and or take into account previous wishes, typically a family carer, and the health and social care providers caring for the person. Best interest decision-making on care preferences at the end of life is complex and can become a significant burden for both family carers and health and social care staff [3–5]. Research evidence highlights the importance of involving family members in end-of-life care decision-making [6–11]. However, a systematic review of families’ experiences’ supporting a dying relative in nursing homes found family carers were disappointed by the limited contact and lack of meaningful communication they experienced with nursing home staff [12]. Families who are not given an opportunity to discuss their relative’s illness, prognosis, and treatment suffer uncertainty about phase of illness and nearness of death, have difficulties with decision-making and can feel unprepared for their relative’s death [12]. Nursing home staff that lack the skills and mechanisms to engage in end-of-life care conversations with family carers may experience moral distress [5]. Further, poorly conceived decision-making place the person living with dementia at risk for inappropriate care [13].

There is evidence that nursing home staff are reluctant to discuss end-of-life care. However, there is evidence to suggest that training increases both competence and confidence in this area [14]. Identified training needs for nursing home staff include understanding the progression of dementia, palliative and holistic care; and, improving communication with residents and family carers [14]. A recently completed systematic review highlighted that nursing home structures and staff play an important role in the successful implementation and adoption of innovations such as the intervention to be examined in this proposed study of the Family Carer Decision Support intervention [15].

The aim of the Family Carer Decision Support intervention is to inform family carers on end-of-life care options for a person living with advanced dementia in nursing home settings. The benefit for family carers is that they can better understand the risks and benefits of care options, actively participate in shared decision-making with health care providers and work together to develop an advance care plan. The intervention involves family carers engaging with two components – an information booklet on end-of-life care options for a person living with advanced dementia called the Comfort Care at the End of Life for persons with Alzheimer’s Disease or other Degenerative Diseases of the Brain followed by a structured meeting with nursing home staff to develop an advance care plan.

The ‘Comfort Care” booklet’ is intended to provide family carers with information so that they can better understand the risks and benefits of care options and the opportunity to actively participate in decision-making. It provides information on the trajectory of the disease, clinical issues, decision- making processes, and symptom management. The booklet has shown evidence of high levels of acceptability among family carers and healthcare providers [16–19]. The information resource is then formally discussed at a structured family meeting, known as a family care conference, including a trained nursing home staff person, family carer(s) and significant others as identified by family carer(s).

This approach to advance care planning has been subject to previous research. The initial Family Carer Decision Support study employed a cluster randomized control trial involving 24 dementia care nursing homes located in Northern Ireland. A trained facilitator external to the nursing home delivered the Family Carer Decision Support intervention. The Family Carer Decision Support intervention demonstrated statistical significant impact in reducing family carer decision uncertainty on establishing care preferences at the end of life and improved family carer satisfaction on quality of care [20]. This implementation study proposes to; a) expand the Family Carer Decision Support intervention into nursing homes; b) extend its application internationally; and, c) train nursing home staff to deliver the Family Carer Decision Support intervention rather than a trained external facilitator.

### Project aims and objectives

The study aims to develop implementation guidance for the Family Carer Decision Support intervention by identifying the facilitators, barriers and resources needed to integrate the Family Carer Decision Support intervention into routine nursing home practice.

### Objectives

1. To develop a staff training module to support staff to deliver the intervention.
2. To develop information resource for family carers on advanced dementia symptoms at the end-of-life, including a question prompt list.
3. To identify the facilitators and barriers to implementing the Family Carer Decision Support intervention into nursing homes.
4. To identify the resource use and costs associated with the successful implementation of the Family Carer Decision Support intervention in nursing homes.
5. To assess the impact of the new model of delivery for the Family Carer Decision Support intervention on both family carers and nursing home staff across different sites and countries.
6. To develop implementation guidance to facilitate use within nursing homes internationally.

## Methods/design

### Design

This research will employ a multiple case study design where a nursing home will be the unit of analysis or ‘case’ [21, 22]. A case study design will allow us to understand the implementation process for the Family Carer Decision Support intervention and to identify the factors which determine how well the intervention will work. We will use a formative design, generating hypotheses about the mechanisms that will lead to successful implementation, testing and refining these hypotheses by introducing the intervention into the nursing homes sequentially (where possible). A sequential approach will allow the experiences and learning from implementing in the first wave to influence implementation approaches taken in the second wave.

The RE-AIM framework will guide the evaluation [23–25]. A major feature of RE-AIM is that it shifts the focus from effectiveness trials to longer-term effectiveness in a real- world setting. The dimensions of the framework are as follows:

- Reach, the proportion and representativeness of identified nursing home staff and family members who access the online training resource and review resource features
- Effectiveness, the impact the advance care planning intervention has on staff and family members knowledge, decisional conflict and attitudes towards the intervention and advance care planning
- Adoption, assess nursing home staff acceptability of the training and family members impression of the suitability of the information resources
- Implementation, assessment of the fidelity of implementing the protocol
- Maintenance, determine whether nursing home managers and staff wish to continue the intervention

### Case sampling and selection

This protocol refers to nursing homes where residents require 24 h nursing care, providing a high level of care involving nursing staff on site. As part of a multiple case study approach, we plan to enrol 16 case study sites across 6 countries (Canada *n =* 2 Republic of Ireland = 2, three regions in the UK *n =* 2 each, The Netherlands *n =* 2, Italy *n =* 2 and the Czech Republic *n =* 2) to reflect the range of characteristics in each national and local context. Therefore across partner countries there will be a high degree of variability in terms of nursing home sizes, structure, staffing and other organisational and cultural components. Recruiting international nursing home sites with such a diverse range of characteristics and contexts will allow researchers to understand more fully how and under what conditions implementation and positive outcomes are supported. Our international team of investigators and advisors, as part of the mySupport study, will provide input into case study site selection to ensure that our sites reflect the type of variability and diversity that exists across the participating partner countries.

### Case sampling

Sampling is important at two levels in this project: a) Case sampling (e.g. variability of geography, country, type and profile of home) b) Sampling within the case which includes sampling participants and sampling other data sources.

### Participant groups within case

We will recruit and collect data from family carers, nursing home staff and, if appropriate, community health care providers who link to the nursing homes (e.g. GPs, social work, palliative care nurses). We will recruit nursing home staff who will be recruited to deliver the intervention and those who are not involved with the intervention. Nursing home staff delivering the intervention will be known as ‘internal facilitators’, and they will be supported with training and on-going bespoke support by one external facilitator for each site. We anticipate recruiting up to three internal facilitators for each site. However, given nursing homes differ in size, culture, staffing, scale and reach in respective partner countries, we anticipate a high degree of variability for sample sizes for all participant groups in each national context. Subsequently, sample sizes in each site will be determined by what is appropriate in each national, local and site context.

### Recruitment of external facilitator

External facilitators will be identified by researchers in each locality. They will be asked to complete the response slip and consent form and return them to the research team in either the pre-paid envelope provided or via email, if they are interested in taking part in the research.

### Inclusion criteria

- Be a trained facilitator with experience in delivering training to Health Care Professionals in a nursing home setting.

#### Recruitment of nursing home staff

Nursing home managers of sites that match our criteria will be approached and invited to participate in the study. The nursing home manager of each of the eligible participating nursing homes will then identify other eligible nursing home staff (i.e. registered nurse or health care assistant) at each facility who meet the inclusion criteria and either post/email them an information pack to include a letter of invitation, Participant Information Sheet, consent form and response slip. They will be asked to complete the response slip and consent form and return them to the research team in either the pre-paid envelope provided or via email, if they are interested in taking part in the research. The researchers contact details are on the PIS should the nursing home staff need any further information prior to agreeing to participate. One to two weeks after the information packs have been given out, the nursing home manager will remind staff about the research and that if they are interested to follow up with the researcher or post back/email the response slip and consent form within one to two weeks.

For those nursing home staff who return a response slip and consent form indicating they are happy to participate in the research, the research team will follow up with them, answer any questions and if they are still agreeable to participation, arrange the interviews.

#### Inclusion/ criteria for nursing home staff (internal facilitators)

##### *Inclusion criteria*

- Hold a nursing qualification and employed by the nursing home as a nurse

#### Inclusion criteria for nursing home staff (not delivering the intervention)

- Work in the nursing home in any care related capacity with residents

#### Recruitment of family members

The nursing home manager(s) and other relevant individuals will identify eligible family members, provide a brief introduction to the study and ask for their consent to be contacted by a researcher. If consent is given, a researcher will make contact and provide an overview of the study, answer any immediate questions and email or post them an information pack (to include a letter of invitation, Participant Information Sheet and consent form). The researcher’s contact details will be on the participant information sheet should the family member need any further information prior to agreeing to participate.

After the information pack has been sent out, non-responders will be followed up by the researcher who will send a second letter of invitation either by post or email asking them to respond.

For those family members who have returned the consent form indicating they are happy to participate, a researcher will follow up with them and answer any questions. If they are still agreeable to participation, the researcher will organise the environmental scan interview at a convenient date and time.

#### Inclusion/exclusion criteria for family members

##### *Inclusion criteria*

- Aged 18 years or older
- Be the individual most involved in the care of the resident as identified by the nursing home manager
- Can understand written and verbal language of site where study is taking place (i.e. English, Dutch, Czech or Italian)

### Exclusion criteria

- Aged under 18 years
- Family members who are unable to communicate through local language

#### Consent process

All staff and family member participants will provide informed consent. The researchers will seek written consent, but if a participant is unable to receive/return consent by mail or email, verbal consent will be audio recorded as a last resort. Participants will be given time to consider taking part in the research from receipt of the PIS and consent form and will have the opportunity to discuss the research and ask questions. During all aspects of the research the researcher will use process consent whereby they will regularly evaluate the comfort of the participant, and if or where appropriate, offer them the option to decline to answer specific questions or terminate their involvement at any time.

If any of the participants become upset, a distress protocol will be followed and support packs will be made available if required to both nursing home staff and family members.

## Patient and public involvement

All partner countries will establish a Public Involvement Panel that will include family carers who will support country specific activities and enable outreach to other stakeholders as required. Baseline interviews will be conducted with all project researchers to explore their: a) views about PPI in research and specifically in this project; b) knowledge about approaches to PPI; c) barriers to PPI participation; and d) perceived potential impacts of PPI in this project. Based on these interviews, project investigators and lay advisors will develop a public engagement plan that accommodates the unique cultural context of each participating country.

## Intervention

The comfort care booklet has been translated into Dutch and Italian language [26] and further adapted to local contexts based on feedback from local healthcare professionals and PPI consultation. We will translate the booklet into Czech. We will produce a question prompt list for each national context, which is set of questions that can be used by family carers as discussion prompts in family care conferences [27].

The family care conference will be held in the nursing home within 3 months of staff training on family care conference procedures. The structure of the family care conference (preparing, conducting, documentation and follow-up) is based on clinical practice guidelines developed for conducting family meetings [28]. In the family care conference the designated staff person in the nursing home will review and discuss the contents of the booklet with family participant(s) facilitating awareness of comfort care practices at the end of life. Family care conference participants will determine the option of follow-up meetings and care planning outcomes.

We will ensure that the Family Carer Decision Support intervention delivered across the different countries aims at achieving the same goals through implementing the same core elements of the intervention for effective delivery. We will distinguish between the core elements of the Family Carer Decision Support in terms of its goals, i.e., the essential items to be included in the materials and the procedures on the one hand, and on the other hand, what items and procedures can be, or should be adapted to fit the local context.

### Implementation training and support for external and internal facilitators

#### External facilitators

We will develop a ‘train the trainer’ model. We will support and train the external facilitators from each country or site who will be responsible for providing training to nursing home staff in each participating country. This external facilitator will also work with the internal facilitators and be responsible for implementing the Family Carer Decision Support intervention in each site.

The train the trainer model will involve e-learning training and ongoing in-person support resources will be developed and piloted prior to implementation in the case study sites. The aim of these resources is to empower and provide external facilitators with the skills required to train and support the internal facilitators.

#### Internal facilitators

We will provide training module for nursing home staff that have been identified to deliver the Family Carer Decision Support intervention. Training content will include: a review of the ‘comfort care booklet’; how to select families, organize and conduct a family care conference; reflection on communication skills required for effective family care conference and documenting family care conference process and outcomes. Trained external facilitators will deliver the nursing home staff training module. External facilitators will also conduct outreach visits to each nursing home for ongoing shared learning, reflective practice as well as supporting staff in employing e- resources (Up to 38 h of contact time per nursing home).

After the training and the internal facilitators will undertake the family care conference with family carers. External facilitators may be directly involved in family care conference as a non-participant observer and or will provide feedback or initial a debrief with internal facilitators where necessary and appropriate. All training and support resources will be developed by the lead study team at Queens University Belfast.

### Data collection

A mixed methods data collection approach is required to capture and make sense of the complexity of implementation. Three phases of data collection include; environmental scan, pre-family care conference data collection and post family care conference data collection.

### Phase 1: environmental scan

First we will conduct an environmental scan where key individuals—(residents (where able), family carers, personal support workers, registered nurses, nursing home managers, and health care providers in the community)—will be interviewed. Questions will be asked on their attitudes, level of support, barriers to implementation and potential cooperation related to the Family Carer Decision Support intervention. The implementation strategy will be modified to address identified barriers from this assessment in each local context. At this stage we will also ask nursing home managers to complete a nursing home profile template for the purpose of gathering information and data on the context of each site.

### Phase 2: pre family care conference data collection

In order to build a family carer profile and to assess the effectiveness of the Family Carer Decision Support intervention, family carers will complete a demographic/visitor profile and outcome measures. These two outcome measures are validated for sensitivity to change and are robust to learning effects:

- Decisional Conflict Scale (DCS). This measures uncertainty and difficulties in the decision-making process [29]. The 16 item version measures five domains: a) uncertainty in choosing options; b) feeling unsupported in decision-making; c) feeling informed; d) decision is consistent with values; and, e) making an effective decision. Items are scored on a 5-item Likert scale. A total score is calculated to assess overall decisional conflict as well as scores for each of the five domains.
- Family Perceptions of Care Scale (FPCS). This 25-item scale was designed to assess family carer perceptions of care provided [30]. The FPCS provides an overall score as well as four subscale scores: a) ‘Resident Care’, which measures family members opinions of care provided to the resident; b) ‘Family Support’, reports on perceptions of nursing home care directed towards family members to assist family members to assist them with decision-making; c) ‘Communication’, concerning the timeliness, comprehensiveness and clarity of the communication between staff and the family member; and, d) ‘Rooming’, assessing perception of appropriate placement of the resident in the facility.

In order to build to a profile of nursing home residents for whom an advance care plan will be developed we will also collect data on their service usage and severity of dementia. The Client Services Receipt Inventory (CSRI) will be used by the nursing home manage in the chart review to record health and social care usage. The nursing home manager will also use the Functional Assessment Staging Tool (FAST) to measure the resident's progression of dementia pre and post intervention.

### Phase 3: post family care conference data collection

Phase 3 will take place approximately 6–8 weeks after phase 2. We will collect post family care conference data for the DCS, FPCS, CSRI and FAST measures. We will undertake follow up interviews with external facilitators, internal facilitators, nursing home managers, other staff not delivering the intervention and family carers to further explore barriers and facilitators to implementation.

### Economic aspects of implementation

An important feature of this evaluation will be to understand the level of resource available and required to permit the implementation of the intervention in each site and country. The inputs used to implement the intervention will be collected and reported on a per-site, per-country basis, and indicative costs calculated. The interviews in phase 1 and 3 will be used to better understand the factors that drive resource use and variation between care homes and partner countries.

### Data collection delivery methods

The methods of delivery for data collection will be flexible in order to accommodate and be sensitive to the needs of the different participant groups and individuals in this study. It is important to be flexible given each individual will be participating in a different regional and national context, and possibly with Covid-19 social distancing measures in place in nursing homes. For example, interviews in the study may be undertaken in-person, by telephone or on MS Teams (GDPR compliant). Questionnaires will be able to be completed in-person, over the telephone or returned by post.

### Data management and analysis

Within each nursing home, data will be analysed a) within data set (nursing home), followed by a b) cross-case analysis. The domains; Effectiveness, Adoption, Implementation, and Maintenance, will be assessed through quantitative descriptive indicators and qualitative interviews with both nursing home staff and family members.

All interviews will be digitally recorded and transcribed verbatim. We will analyse qualitative data in NVIVO and follow the framework analysis style outlined by Ritchie and Spencer [31]. This will be informed by the analysis of qualitative data, for which we will develop codebooks for each participant group and time point in the study. These codebooks will be developed in collaboration with researchers from each partner country following analysis of the first two interviews in each country. The analytical process using the codebooks will drive two higher level analytical processes. Firstly, they will be used in conjunction with the nursing home profile to develop an individual site template to develop a case-by-case analysis. This will also include the quantitative data. Descriptive statistics will be used to analyse the quantitative data collected from the DCS and FPCS and will be used to triangulate the qualitative data. Secondly, the codebooks and the individual site templates will inform the development of a cross-case framework to further summarise and synthesise data by generating themes, patterns, and interrelationships in an interpretive fashion.

During the analysis we will compare the pooled outcome measure data from the current adapted Family Carer Decision Support intervention to the outcome measure data that was collected as part of the earlier cluster randomized control trial involving 24 nursing homes located in Northern Ireland [20]. This will determine how the adapted implementation approach for scaling up the intervention compares to the original implementation approach.

### Ethical and governance issues

To minimise participant burden, the total duration of interviews will be limited to a maximum of 60 min. Interviews will be undertaken by experienced researchers in the field of nursing home research.

Participation will incur time and some interviews may cover emotional and distressing issues. Participation is voluntary and any participant can choose to end their involvement in the study without needing to give a reason why. However, we also anticipate several benefits from taking part in this study. It is anticipated that nursing home staff and family members will be more knowledgeable and feel more prepared in having advance care plan conversations.

Involvement in the study will be kept confidential. Participant information will only be accessible to members of the research team. Personal information will be coded with a unique identifier number (a number linked to participants name which only the research team will have access to on an encrypted file). We will keep all information safe and secure on Microsoft Teams (GDPR compliant), and participants’ identities will be anonymised in any publications or other outputs. Digital recordings will be deleted as soon as the transcript is transcribed. All information will be treated as strictly confidential and handled in accordance with GDPR.

### Dissemination and knowledge, transfer and exchange activities

It is envisaged that the study will produce a number of outputs, including: news media; digital media targeted at different stakeholders (such as family members, nursing home staff, policy makers, researchers and academics); conference presentations and webinars; open access publications in academic journals. Implementation guidance will also be written to guide researchers, healthcare staff and policymakers who may wish to implement this intervention or similar in the future.

The guidance will provide coherent and complex information about the most effective way of implementing Family Carer Decision Support intervention in nursing homes, taking into account cultural and health care systems differences across countries.

Communication is a central pillar of this project in respect of implementation delivery and the intervention focus. On this basis, we will develop and implement a knowledge transfer, exchange (KTE) and communication plan and support and develop early career researcher’s in dementia and palliative care research. The knowledge exchange and dissemination plan will be informed by an Evidence-based Model for the Transfer and exchange of Research Knowledge for palliative care research [32]. The plan will outline the content to be communicated to nursing home sites and wider resources to disseminate (end of project outputs, short term outcomes, and long-term impact), map the stakeholders (internal and external); define potential benefits and KTE strategy for early career researchers. The knowledge exchange and communication plan will include the following activities: developing a project website, establishing an early career research committee to share learning across partner countries, a mentoring scheme for early career researchers and knowledge exchange committee to plan activities, develop study newsletters, design project logos and branding.

## Discussion

### Strengths

The protocol takes into consideration social distancing and other relevant Covid-19 infection prevention and control practices. Although this study is not directly focused on the context of the Covid-19 pandemic, the timing of the study means that it will take place when levels of Covid-19 infection could well be significant in each national context. On this basis, we will provide resources/tools and equip nursing homes, their staff, residents and family members during a time when developing an end-of-life care plan or advance care plan has never been more pressing for people.

The research builds on previous evidence of the effectiveness of the Family Carers Decision Support intervention and through a multiple case study design will evaluate the intervention in different national, regional, cultural, social and organisational contexts.

While it is hoped that the intervention resources will impact on policy and practice in the nursing homes that are recruited to the study, the development of implementation guidelines will ensure impact on wider national policy and practice. It is our aim that the resources will be sustainable beyond the duration of the study and this will enable the resources to have a longstanding relevance for future advance care planning practice for staff, family carers and residents with advanced dementia.

### Limitations, challenges and further opportunities

Should this project need to be implemented and delivered using remote and online methods due to Covid-19 restrictions, this is likely to impact on the delivery of the project. This is both a potential limitation, but also an area of interest given that implementing and delivering remotely and online may highlight novel approaches which will make considerable additions to the literature on advance care planning and implementation research in this wider field. Given the intervention will be implemented in six countries, remote and online delivery may be a factor or variable in some contexts and not others. This offers interesting and different implementation contexts, and is congruent with a multiple case study design which will allow us to understand the implementation process for the intervention and to identify the factors which determine how well the intervention will work.

This study will prompt further innovative research on advance care planning and staff education in nursing homes in the context of Covid-19. For example, UK based members of this project team and other colleagues have secured funding for a UK based project that will develop advance care plan training and informational resources for nursing home staff and family carers in the context of Covid-19 [33, 34].

## Data Availability

De-identifiable data and study materials will be shared and requests will be judged on an individual basis. Please contact KB to make a request to access the data.

## Abbreviations

CSRI: Client Services Receipt Inventory
DCS: Decisional Conflict Scale
FAST: Functional Assessment Staging Test
FPCS: Family Perceptions of Care Scale
RE-AIM: Reach, Effectiveness, Adoption, Implementation, Maintenance Framework
